# Supplementary material for: Dynamic Clustering and Scaling Behavior of Active Particles under Confinement
Source: Nanomaterials (Basel). 2024 Jan 9;14(2):144. doi: 10.3390/nano14020144 (PMC10819351; doi:10.3390/nano14020144)
Supplement: Supplementary file 1 [file nanomaterials-14-00144-s001.zip › nanomaterials-2800362-supplementary.pdf]

Supplemental Material

Dynamic Clustering and Scaling Behavior of Active Particles under Confinement

Matthew Becton [1], Jixin Hou [1], Yiping Zhao [2] and Xianqiao Wang [1,*]

[1] School of ECAM, College of Engineering, University of Georgia, Athens, GA 30602, USA; becton@uga.edu (M.B.); jixin.hou@uga.edu (J.H.)
[2] Department of Physics and Astronomy, The University of Georgia, Athens, GA 30602, USA; zhaoy@uga.edu
* Correspondence: xqwang@uga.edu

Methods

DLVO Theory: Depending on the individual particle properties, as well as the dispersion fluid, colloids can aggregate, flocculate, or remain discrete within the fluid environment. Due to the fluid inertia and small length scales, most mathematical models for the base fluid on these scales use the Navier-Stokes equations of state for fluids, assuming incompressibility, and Neo-Hookean or rigid body material for the solid particles; the associated approximations included in these assumptions are favorably validated with experiments [1-4]. DLVO theory, named after Boris Derjaguin, Lev Landau, Evert Verwey, and Theodoor Overbeek, quantitatively describes the aggregation behavior of aqueous dispersions, and as such is very useful for the modeling of colloidal interactions, which generally present long-range repulsion and short-range attraction between dispersed particulates [5-7]. DLVO describes the force between two spheres interacting through a combination of Lennard-Jones and doubly-screened electrostatic interactions, and with a certain amount of modification can also be used to describe nonspherical particles [8]. Due to the doubly-screened electrostatic interactions felt by the particles, aggregation behavior of charged particles is strongly dependent on the screening potential / chemical makeup of the suspending

fluid, with the polar solvent water being the most commonly used [9]. Thus, in this work, DLVO-based interaction potentials are used for colloid-colloid interactions, with a multiparticle collision-based method used to model the solvent.

Stochastic Rotation Dynamics: SRD is a dynamic mesoscopic coarse graining technique which has been previously used to capture the behavior of passive and active colloidal particles under low Reynolds numbers and low to moderate Peclet numbers [10-12]. SRD was developed for cases when fluid dynamics are important, but the detailed chemical properties can be neglected. Compared to DPD, where transport coefficients and effective viscosities can vary by up to 50%, SRD values are generally within 1% of experimental results. The lattice Boltzmann method for solvent particles can be more efficient than SRD, but is also far less accurate in regimes where thermal fluctuations are needed (such as the effect of Brownian motion). Brownian dynamics can be more efficient than SRD, but less accurate if inertia and full hydrodynamics are necessary. The fluid-representative solvent is composed of point masses, and SRD draws from both Monte Carlo and lattice methods with no numerical instabilities. It can also be easily coupled with MD simulations. The SRD method maintains synchronous, discrete-time dynamics with continuous velocities and local multiparticle collisions used to efficiently describe the dynamics of the solvent, while colloidal particles are coupled to the solvent through explicit interaction potentials [10].

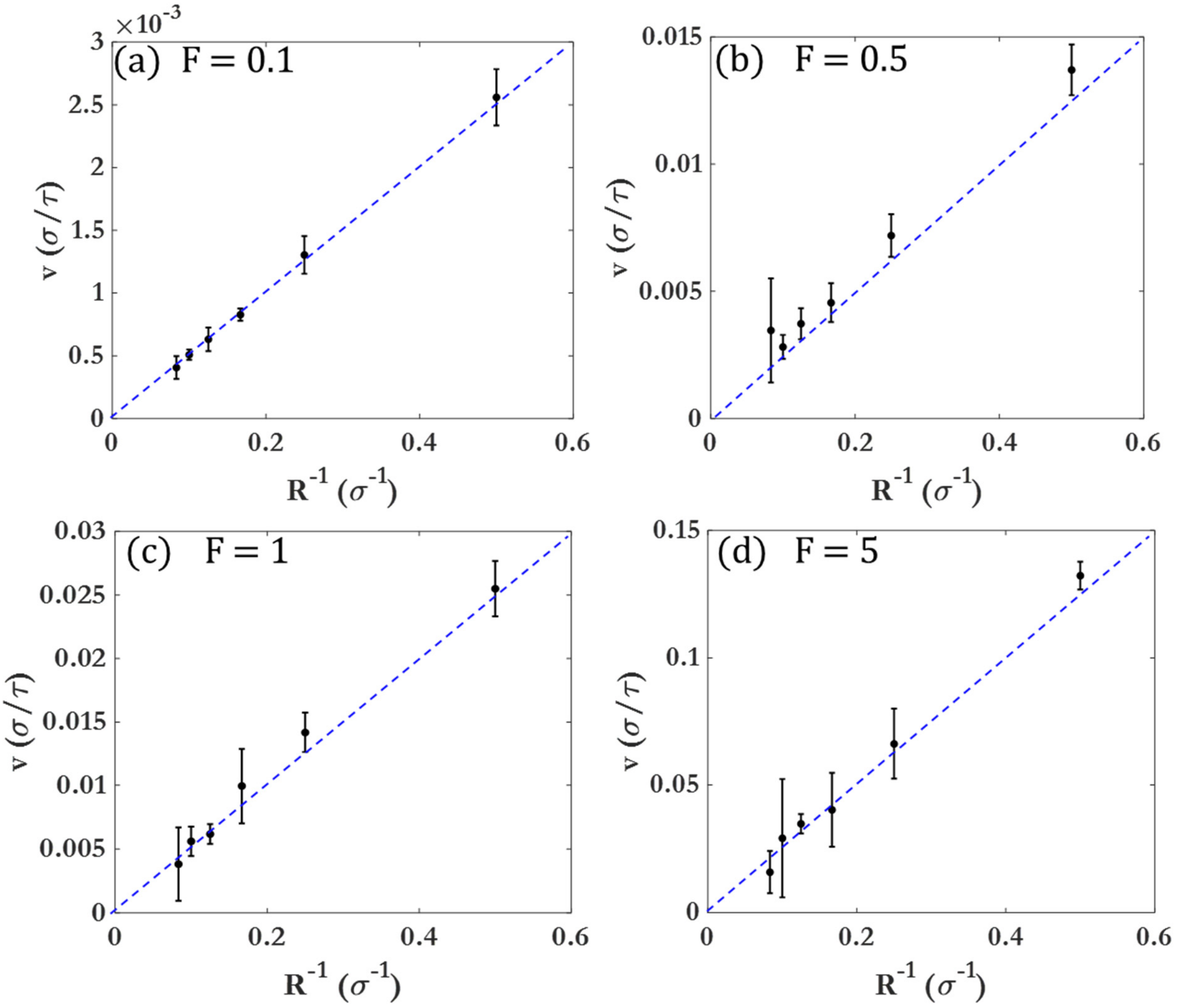

Figure S1: Low Reynolds regime for laminar flow test cases. Cases are for spheres of different radii under driving force (a) F = 0.1, (b) F = 0.5, (c) F = 1, (d) F = 5. The dotted lines are derived from theory.

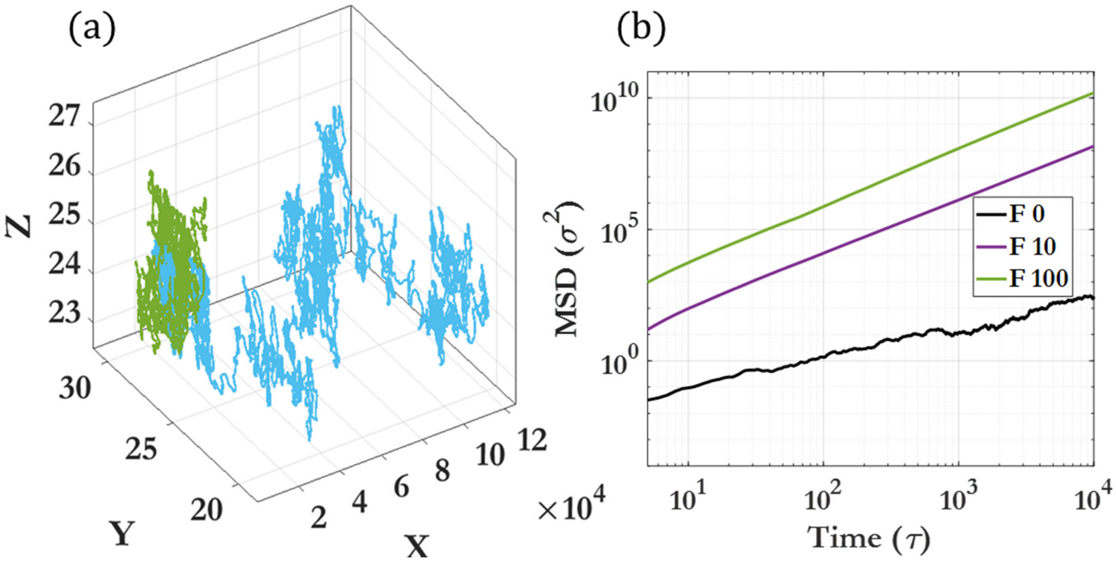

Figure S2: Path of a single bead (a) and MSD (b) comparisons.

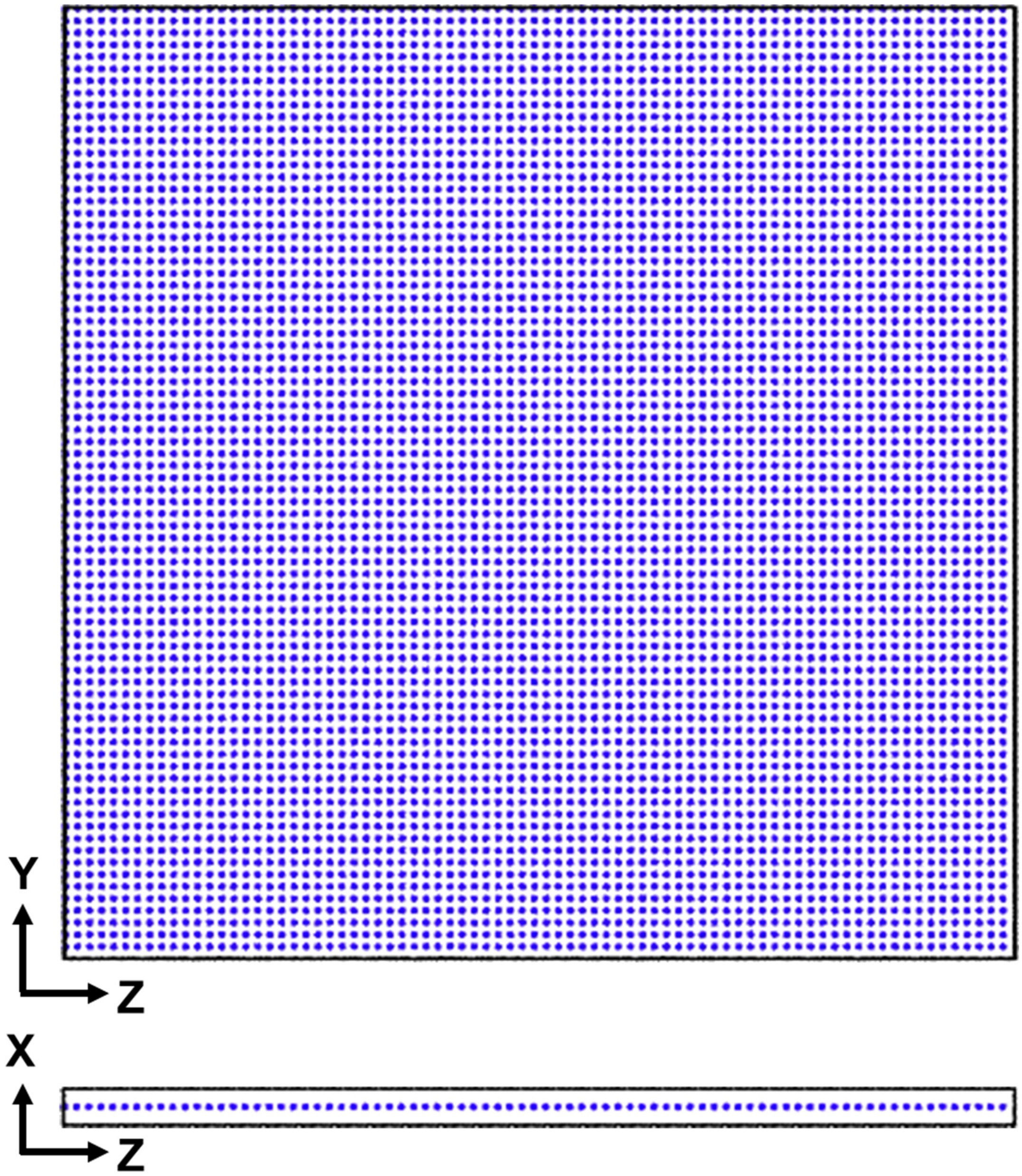

Figure S3: Axes and initial experimental setup for θ = 30%.

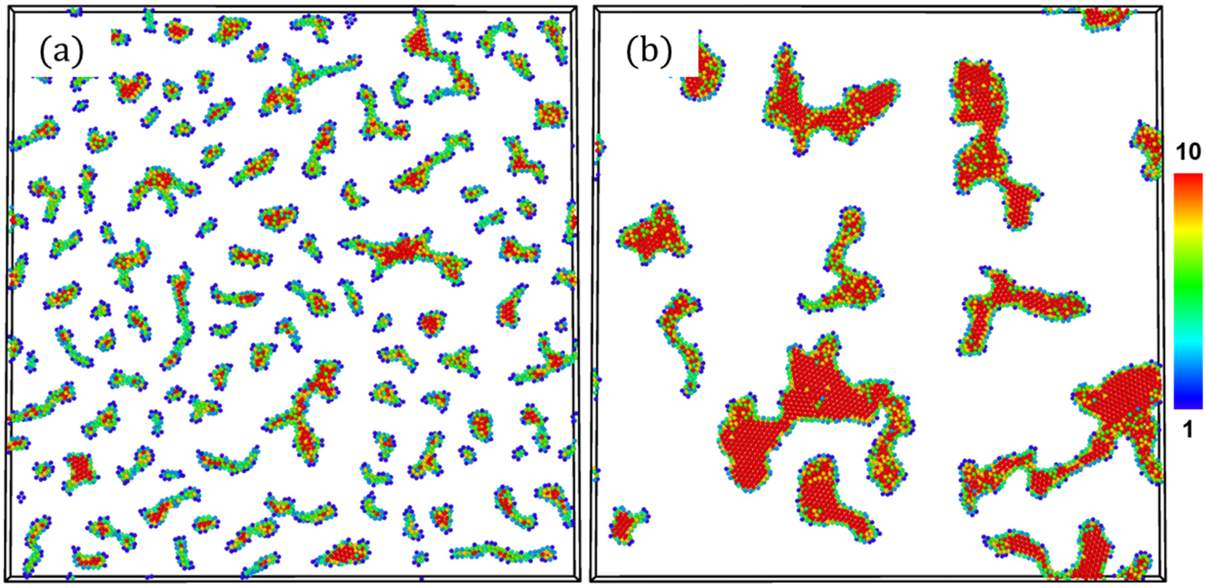

Figure S4: Coordination during clustering for θ = 30% F = 5. (a) τ = 275, (b) τ = 2,500. The color bar indicates coordination of the beads.

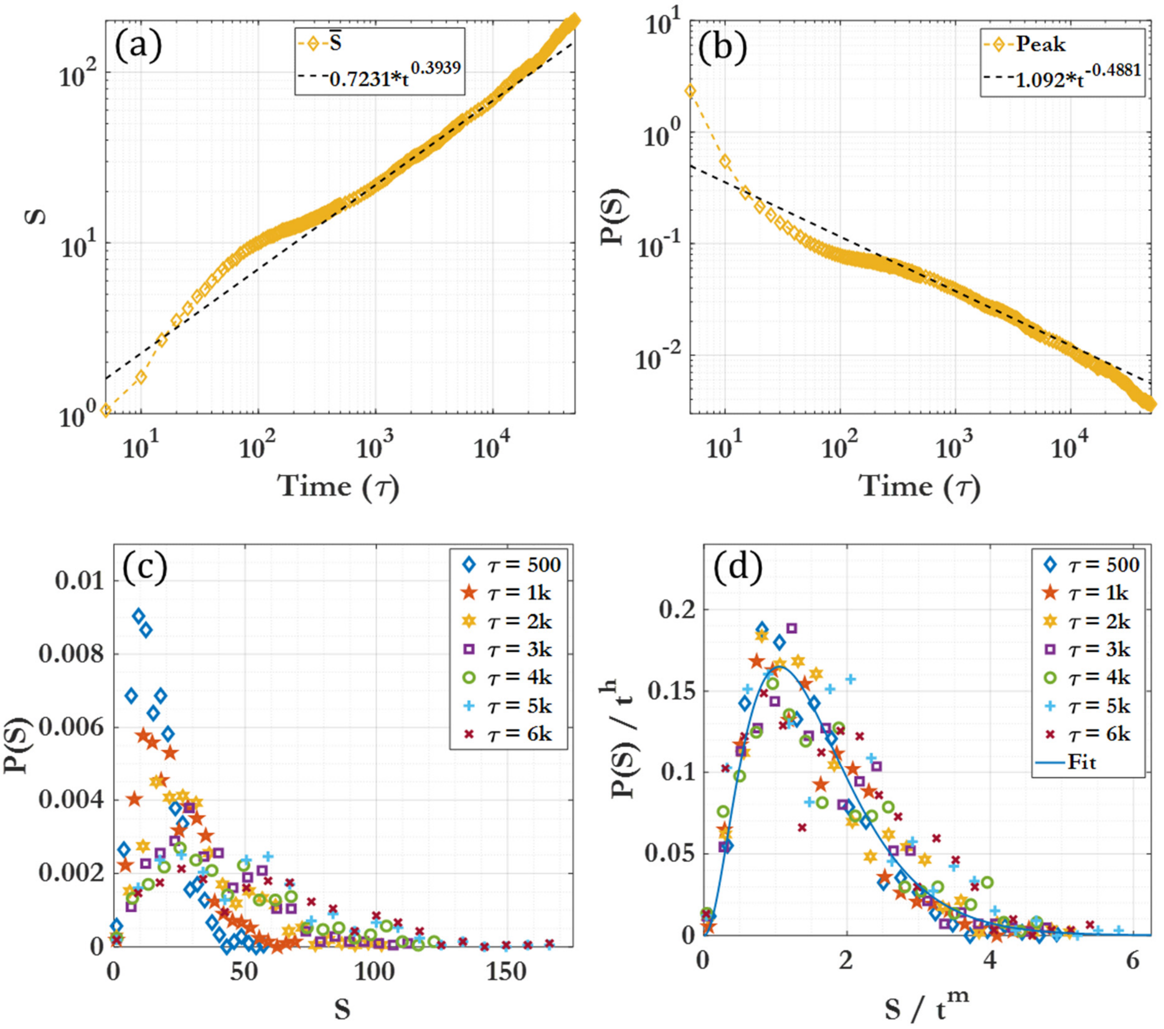

Figure S5: Labeled Regions 4 for θ = 20%, F = 0.5. (a) Mean size fitting, (b) size peak probability fitting, (c) cluster sizes for Region 4, (d) scaled cluster sizes for Region 4. m and h are scaling exponents.

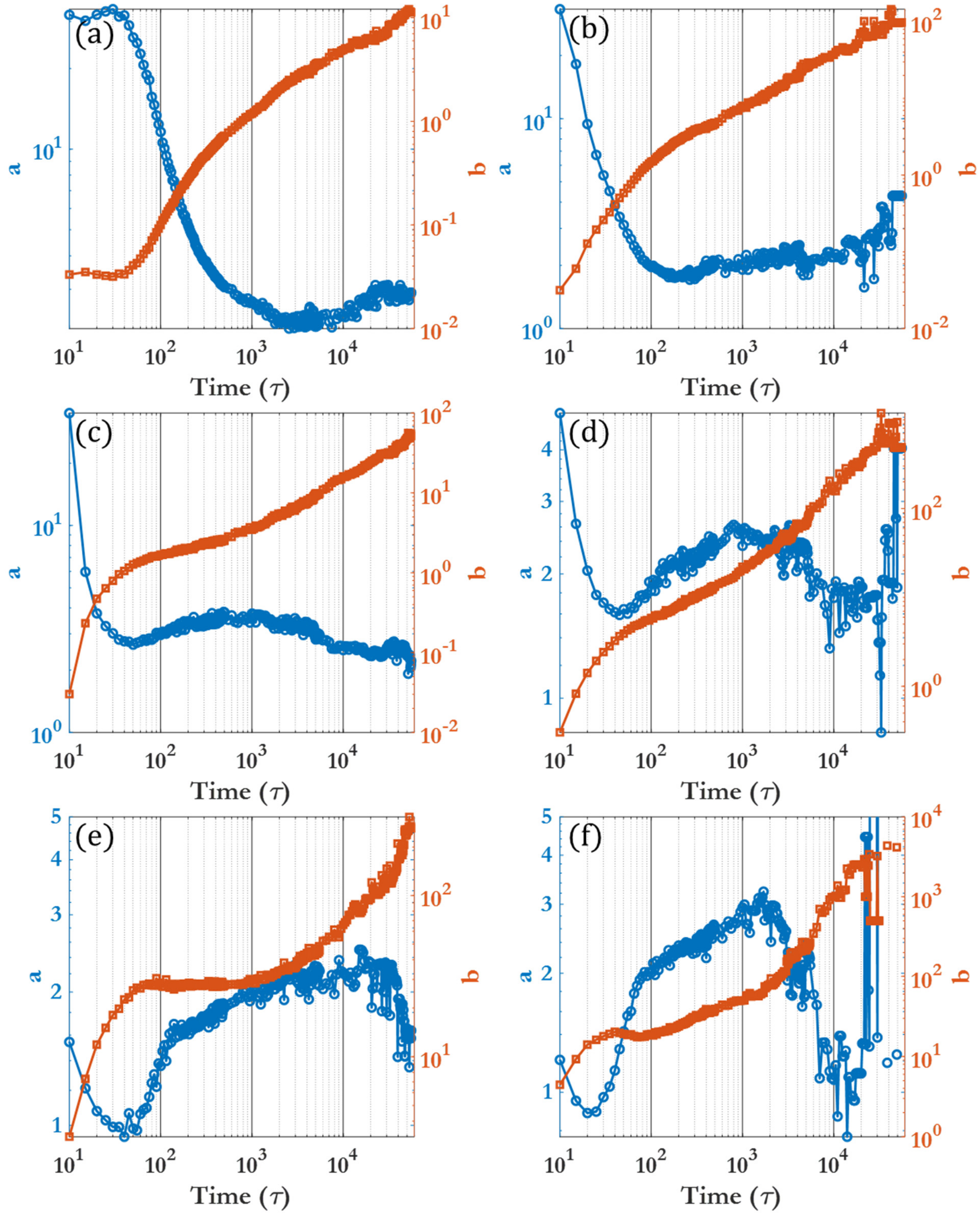

Figure S6: Gamma function a (shape) and b (scale) parameters over time for (a) θ = 5%, F = 0, (b) 5; (c) θ = 15%, F = 0, (d) 5; (e) θ = 30%, F = 0, (f) 5. The solid vertical lines mark the transition between regions.

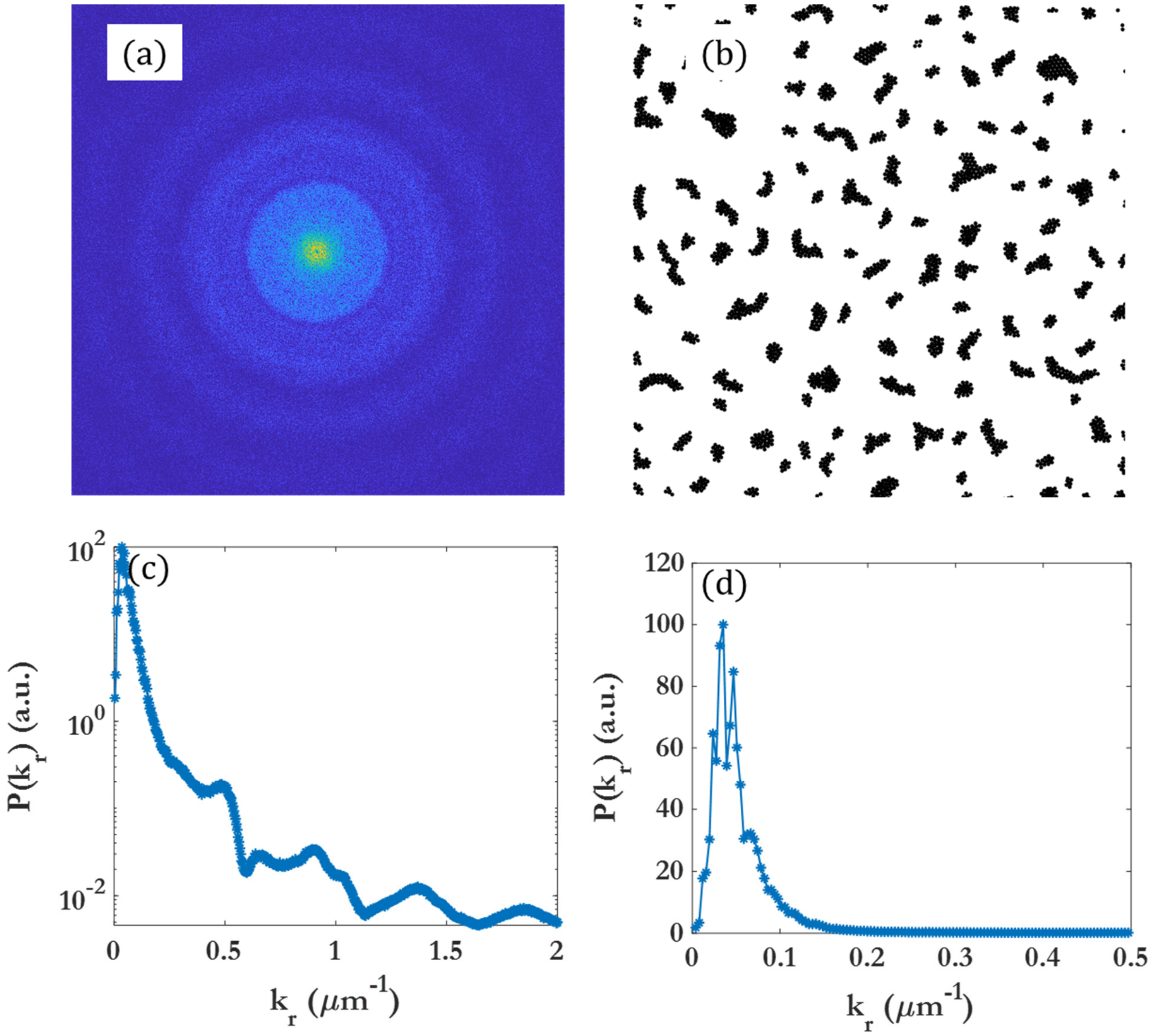

Figure S7: Cluster spatial distributions. (a) FFT of image in (b) for θ = 15%, F =0, t = 5000 τ (c) and (d) show the circularly averaged intensity profile.

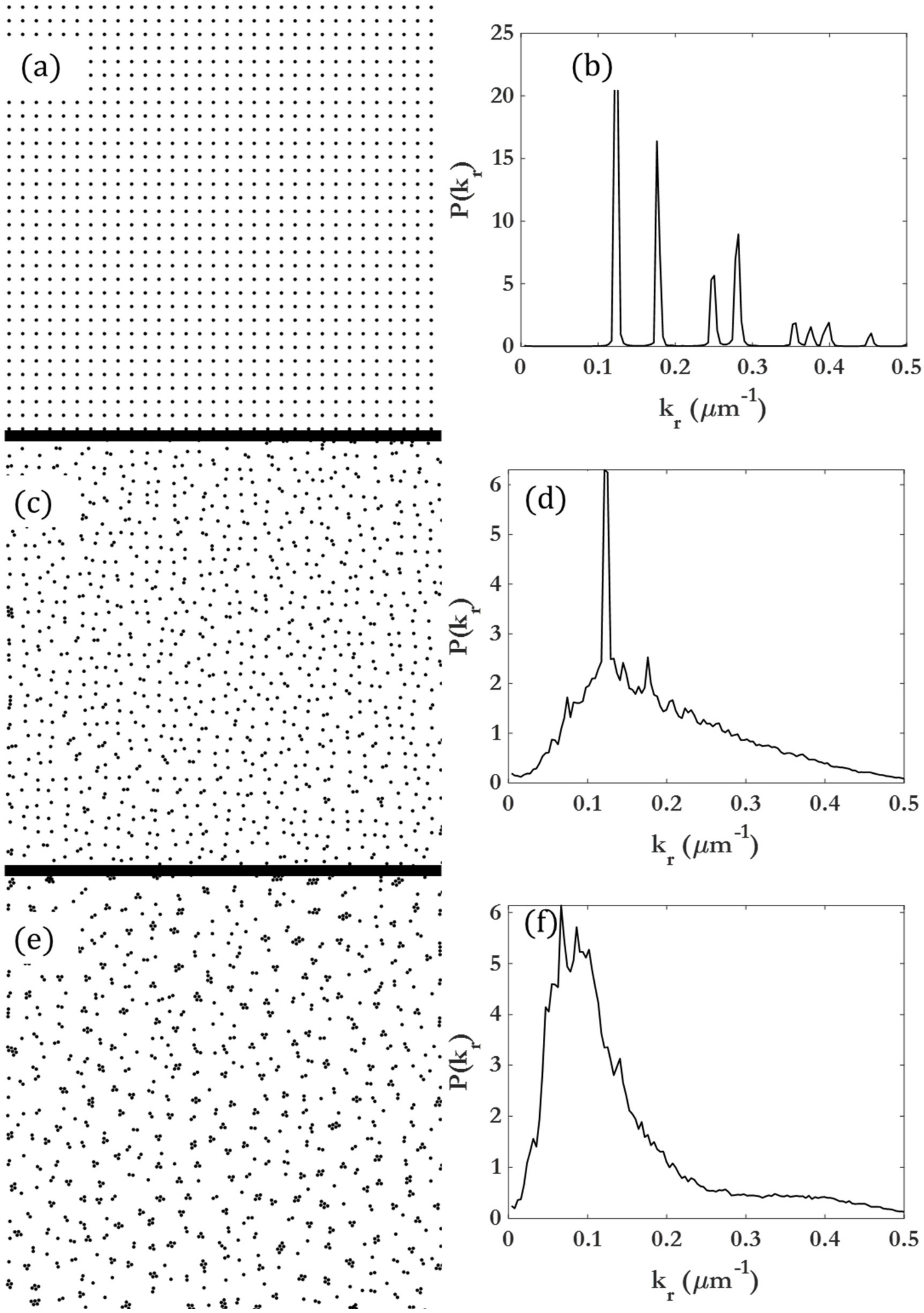

Figure S8: (a, c, e) Cluster distribution and (b, d, e) FFT power spectra showing presence and disappearance of crystalline peaks due to the initial setup geometry for $\theta = 5\%$, $F = 0$ for (a, b) $\tau = 0$, (c, d) $\tau = 100$, (e, f) $\tau = 500$.

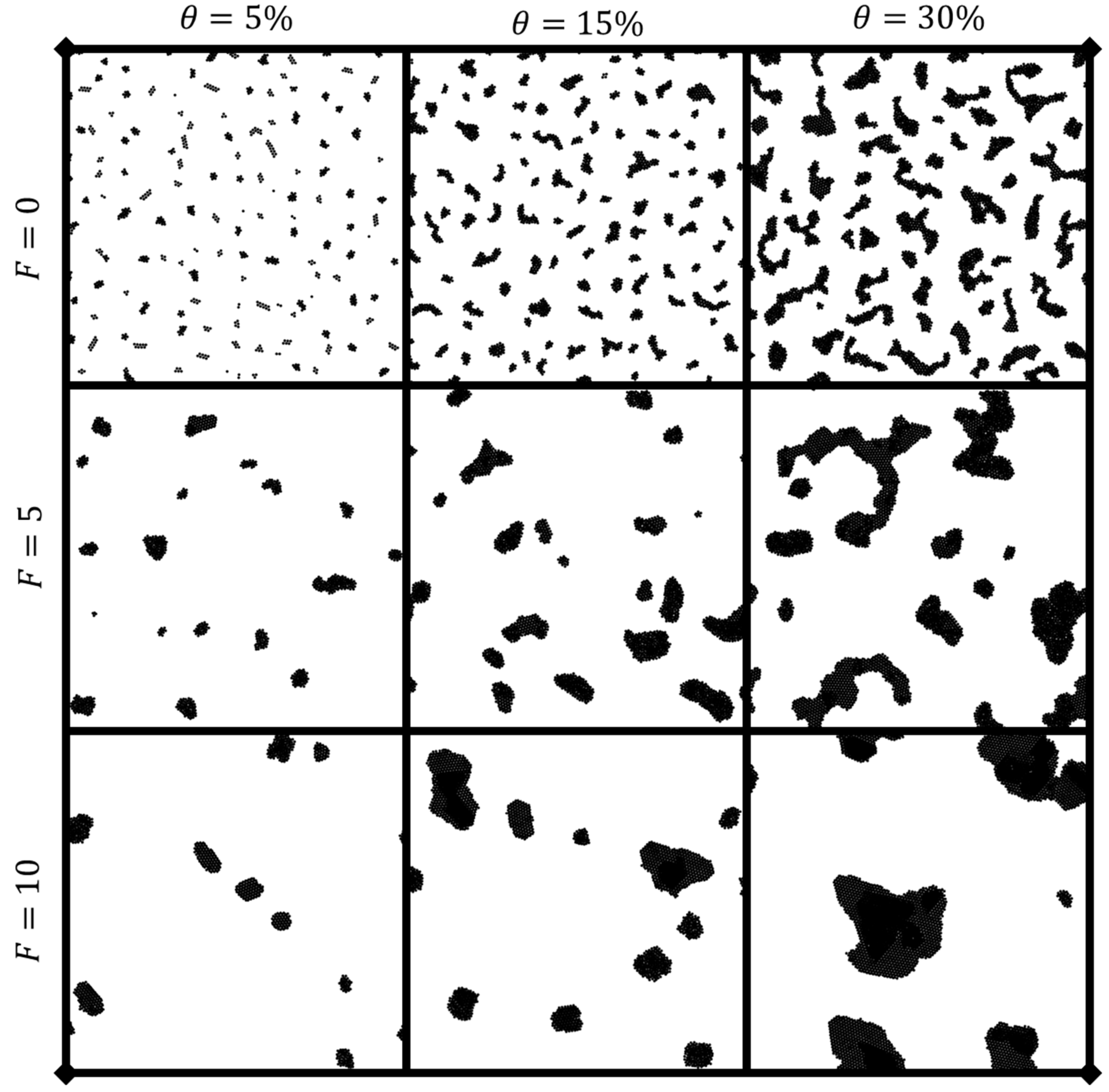

Figure S9: τ = 5,000 for the cases shown in Figure 3.

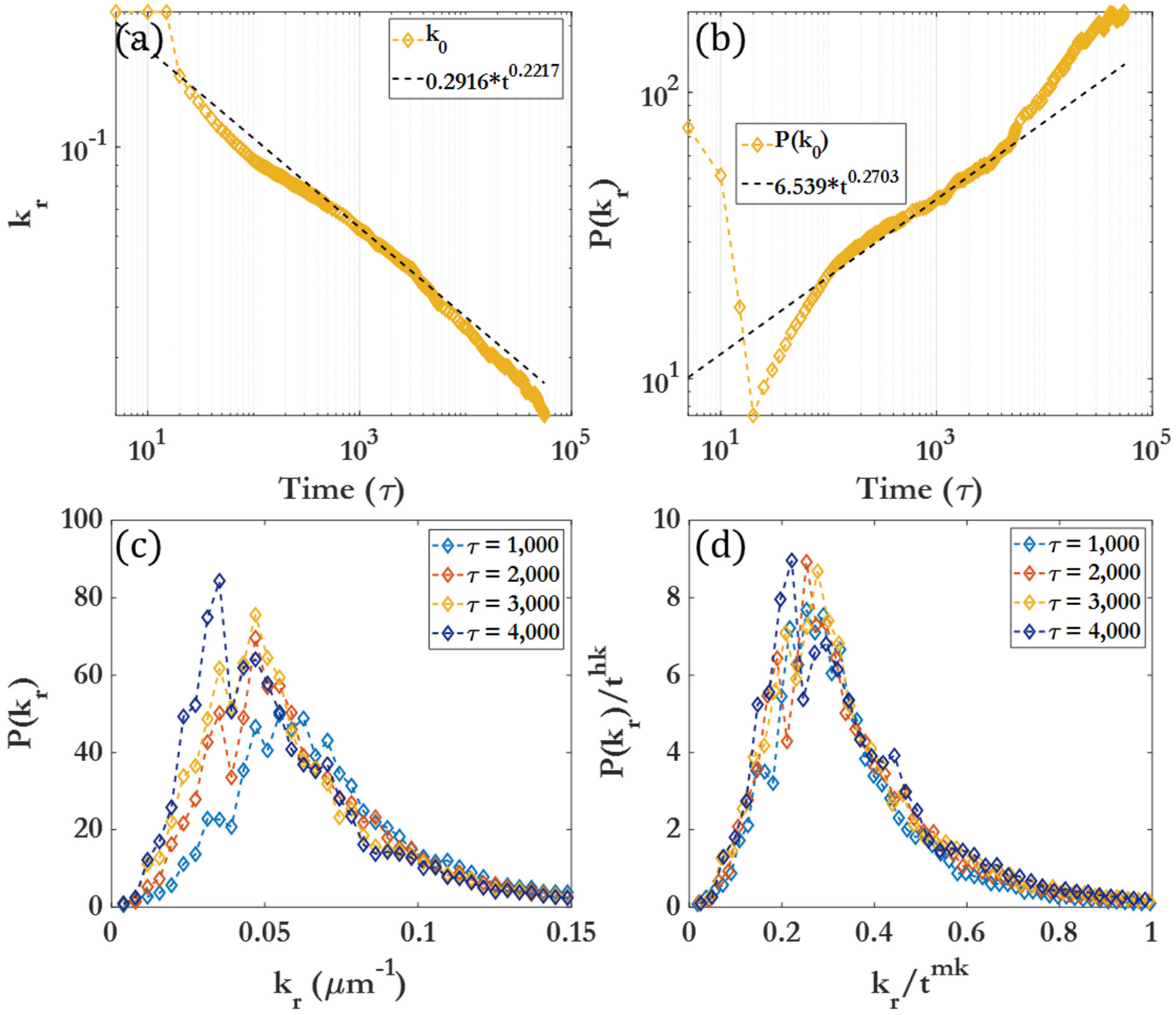

Figure S10: 2D circularly averaged FFT spectra over time for θ = 15%, F =0. (a) The peak center for k0, with fitting line for Region 4; (b) the peak intensity of k0, with fitting line for Region 4. (c) The FFT spectra for t = 1,000 τ to t = 4,000 τ. (d) The same spectra after being scaled by the scaling function for the pertinent region.

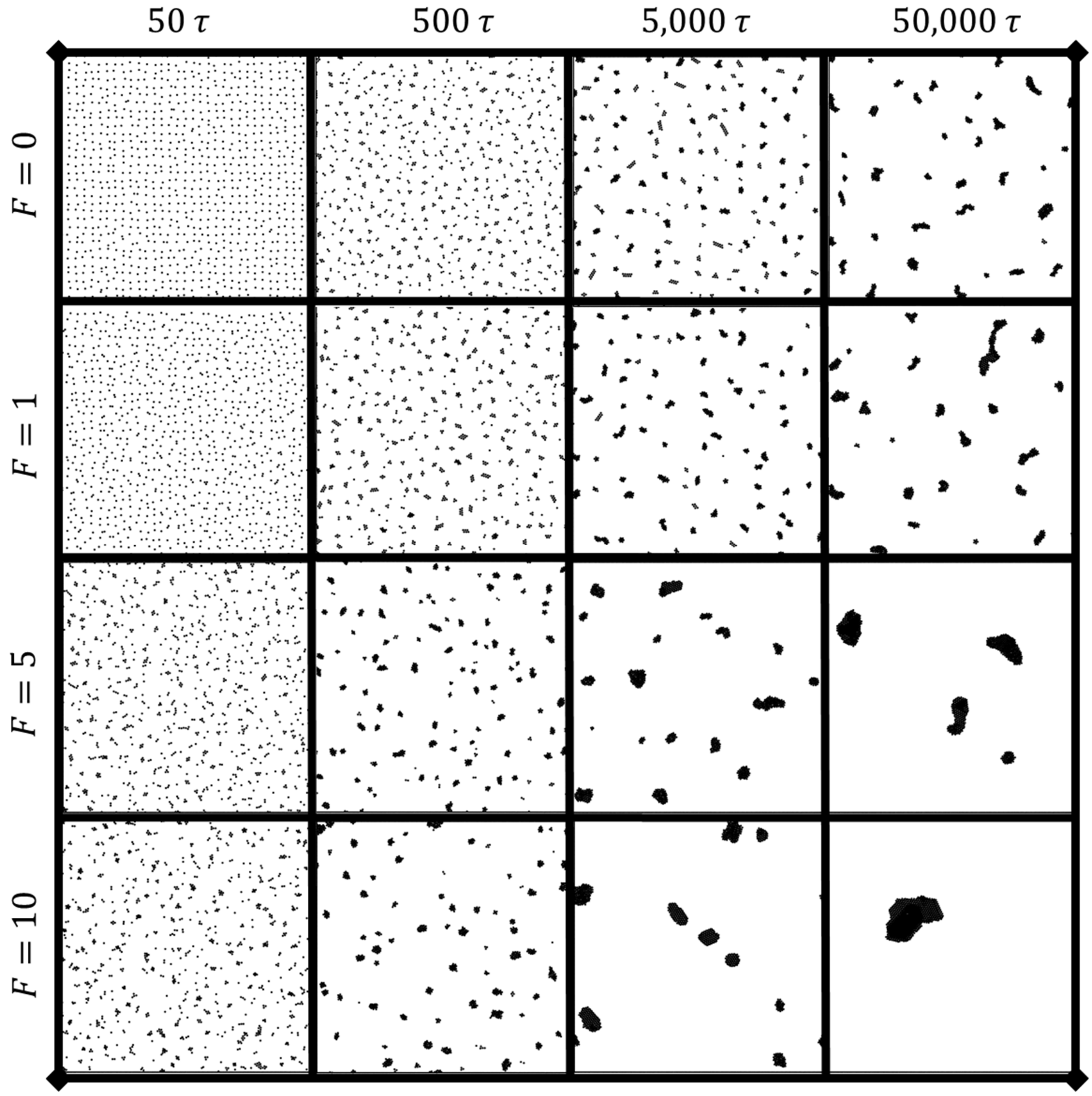

Figure S11: Clustering behavior over time for θ = 5%.

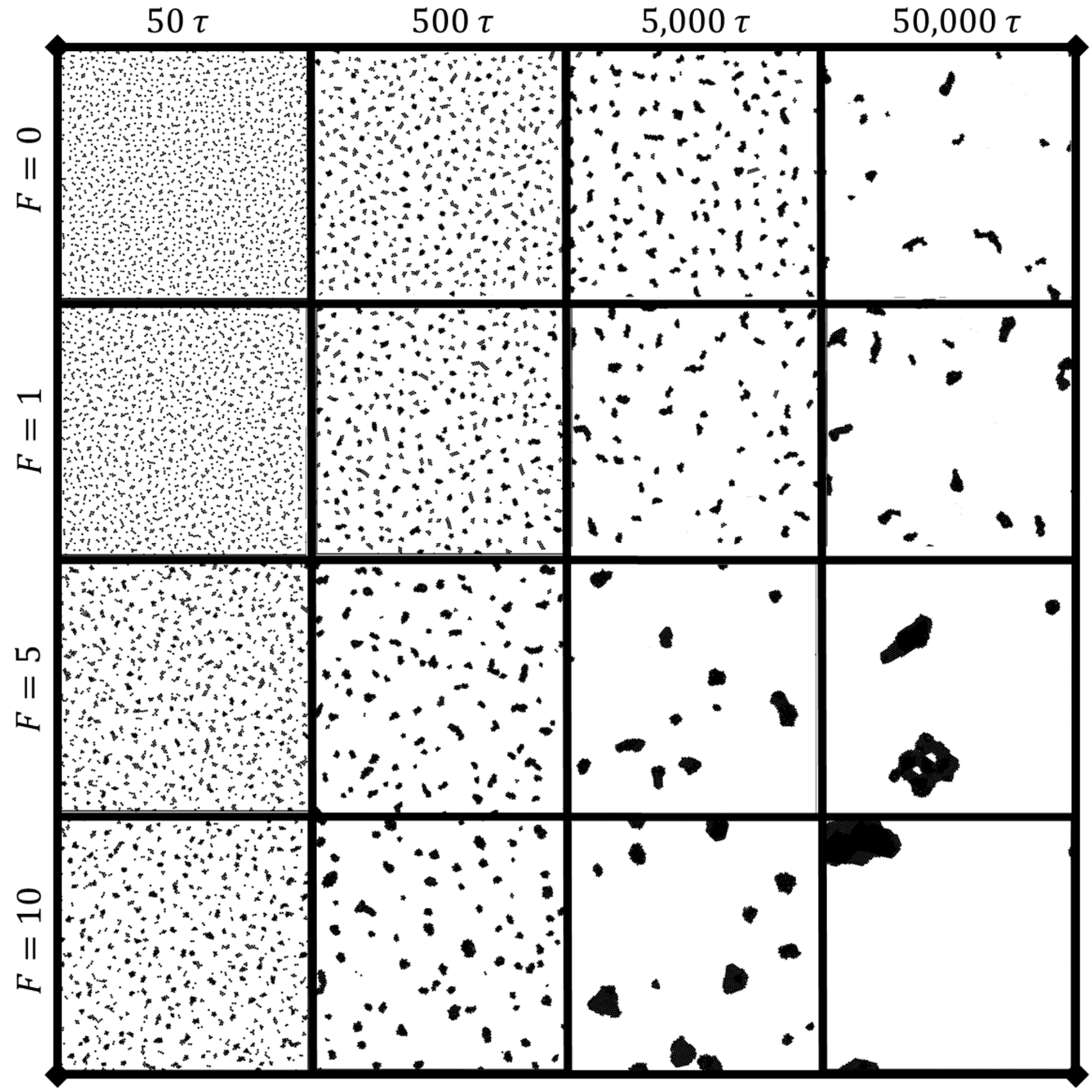

Figure S12: Clustering behavior over time for θ = 10%.

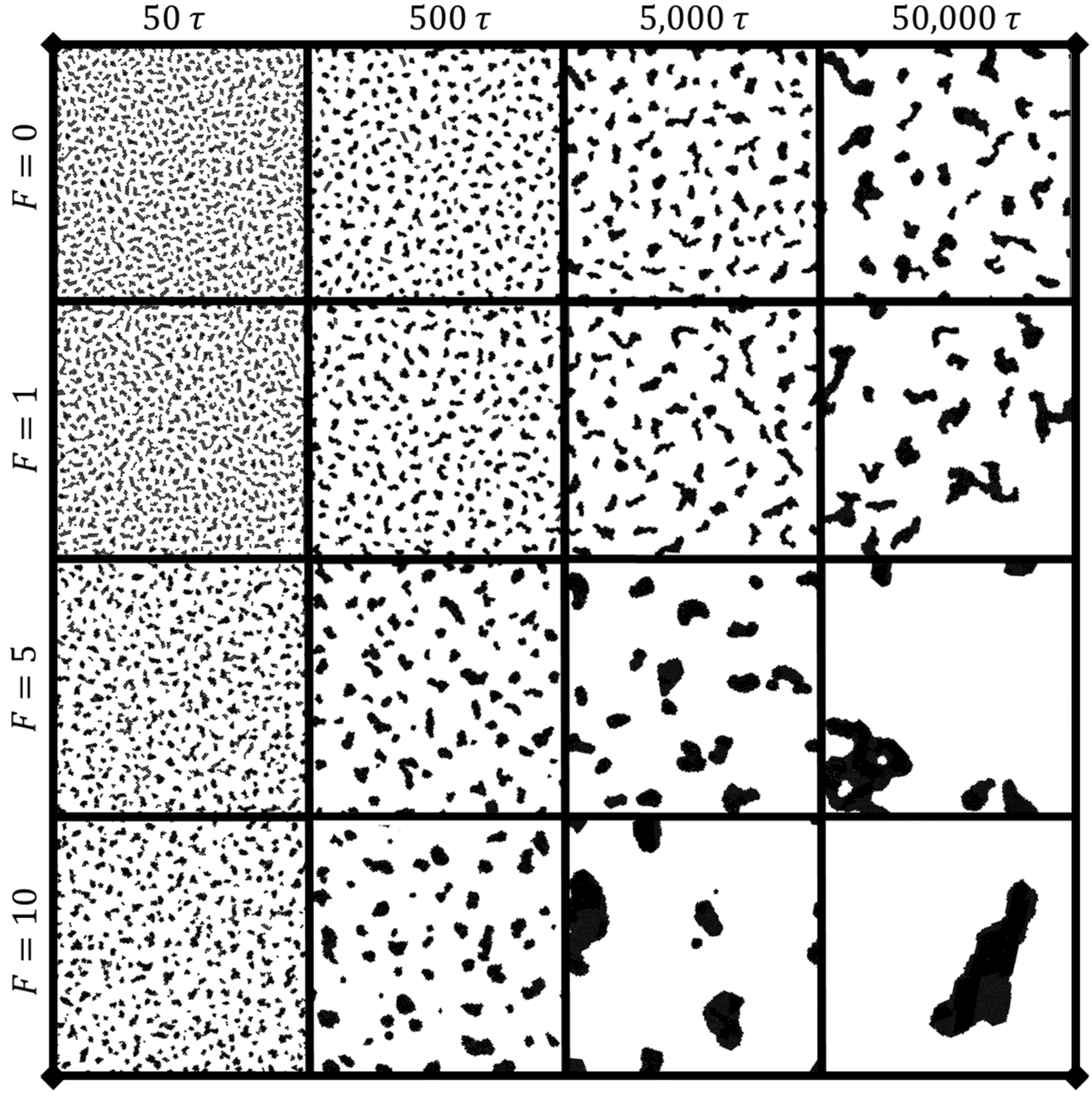

Figure S13: Clustering behavior over time for θ = 20%.

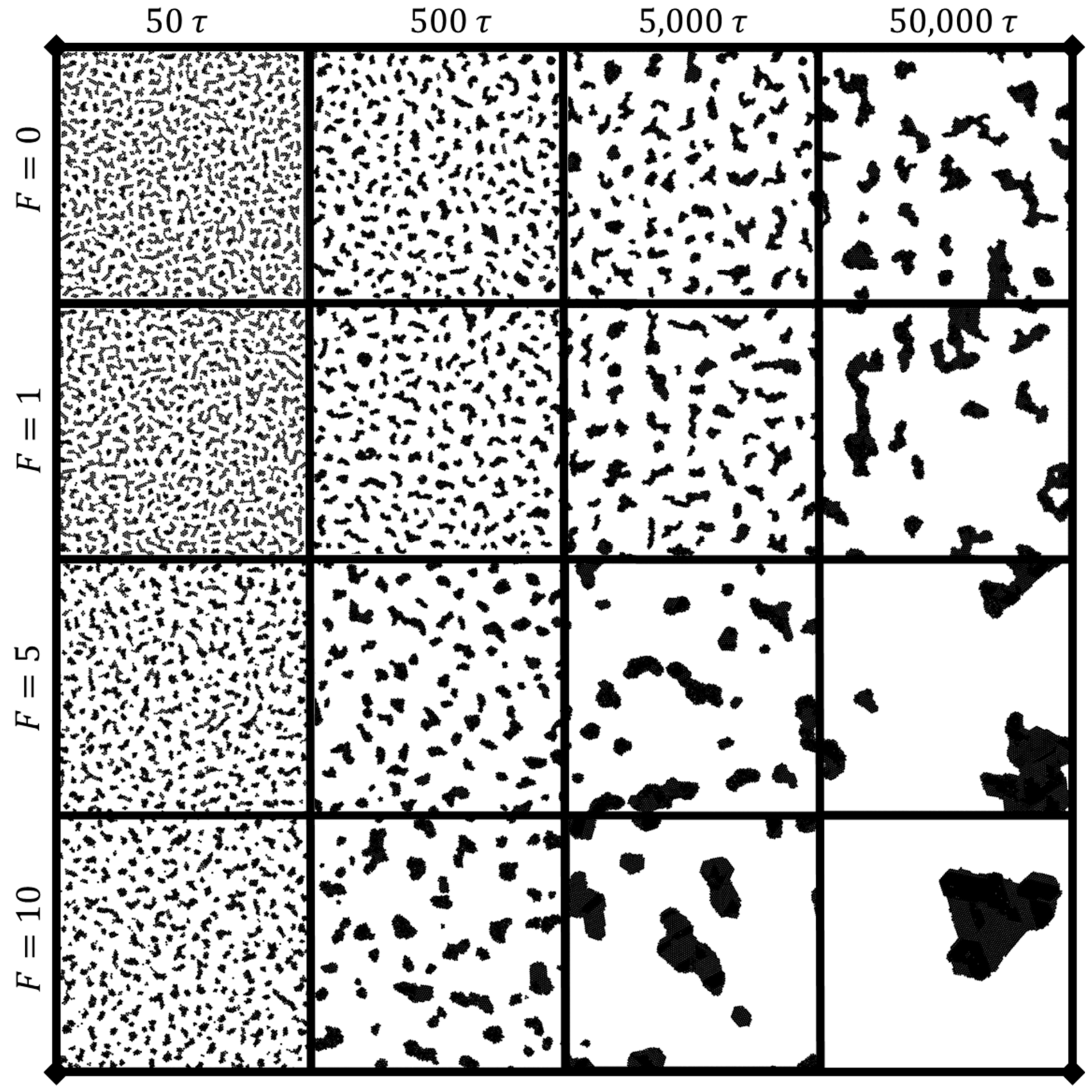

Figure S14: Clustering behavior over time for θ = 25%.

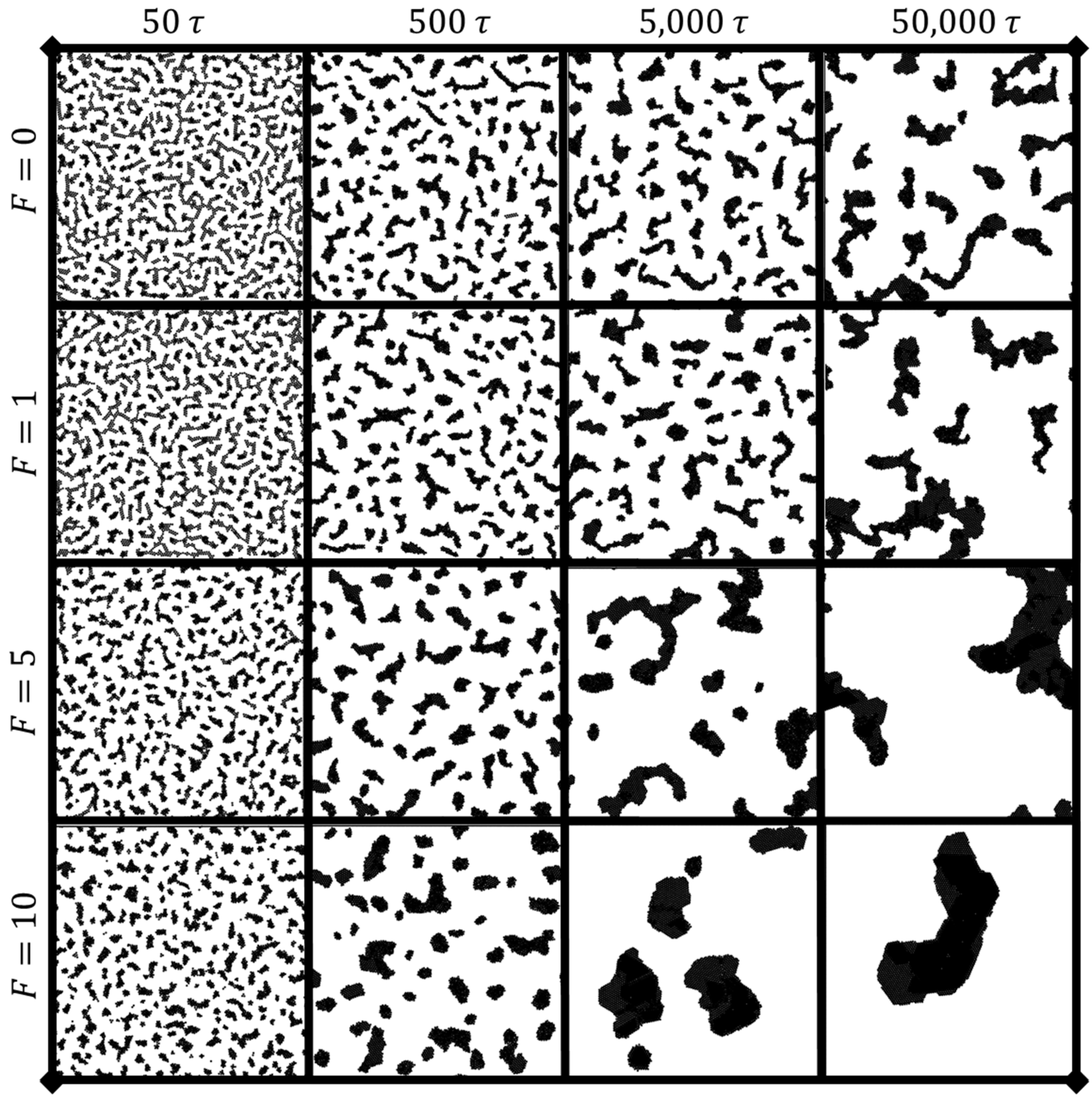

Figure S15: Clustering behavior over time for θ = 30%.

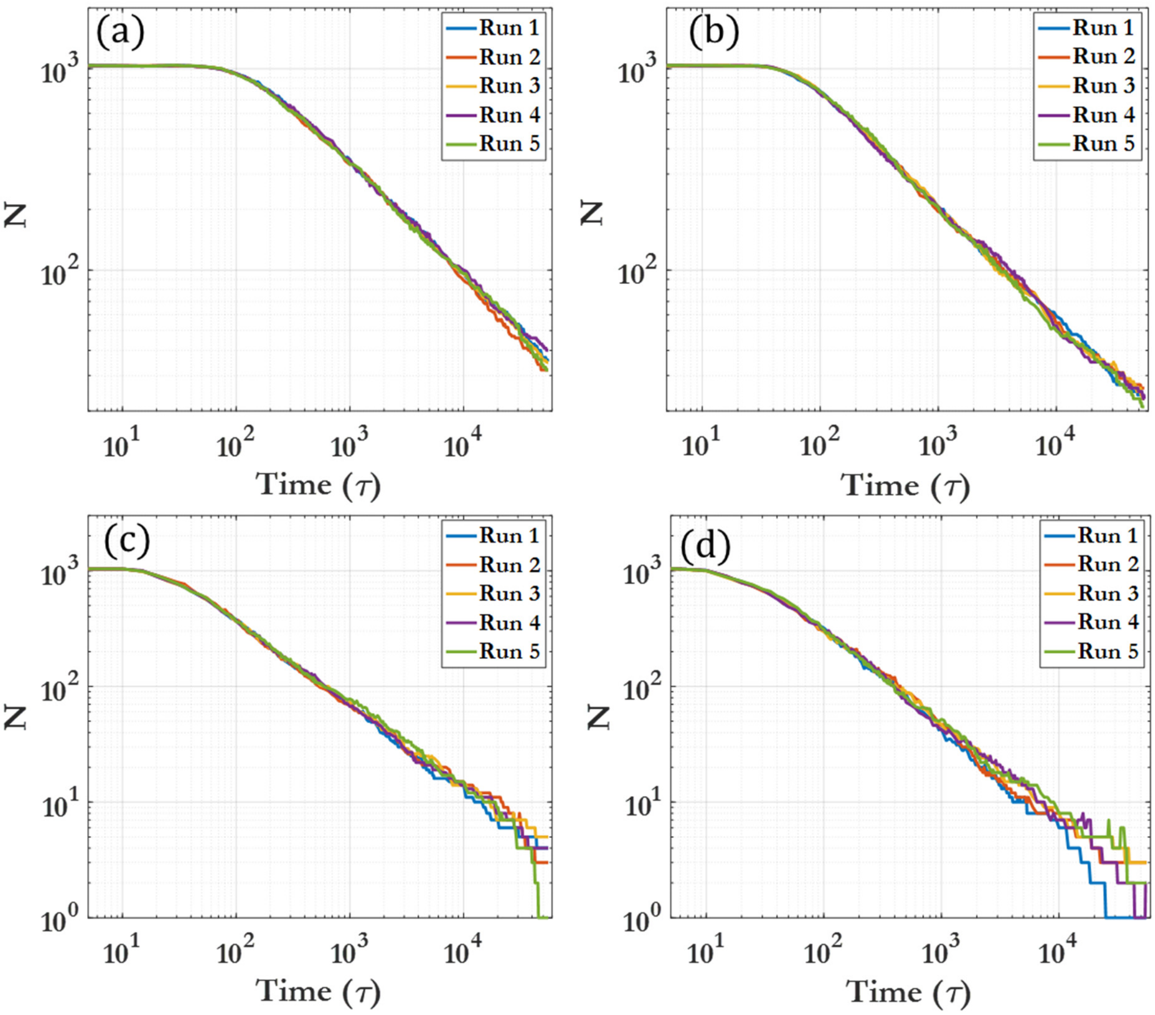

Figure S16: Different runs for θ = 5%, (a) F = 0, (b) F = 1, (c) F = 5, and (d) F = 10.

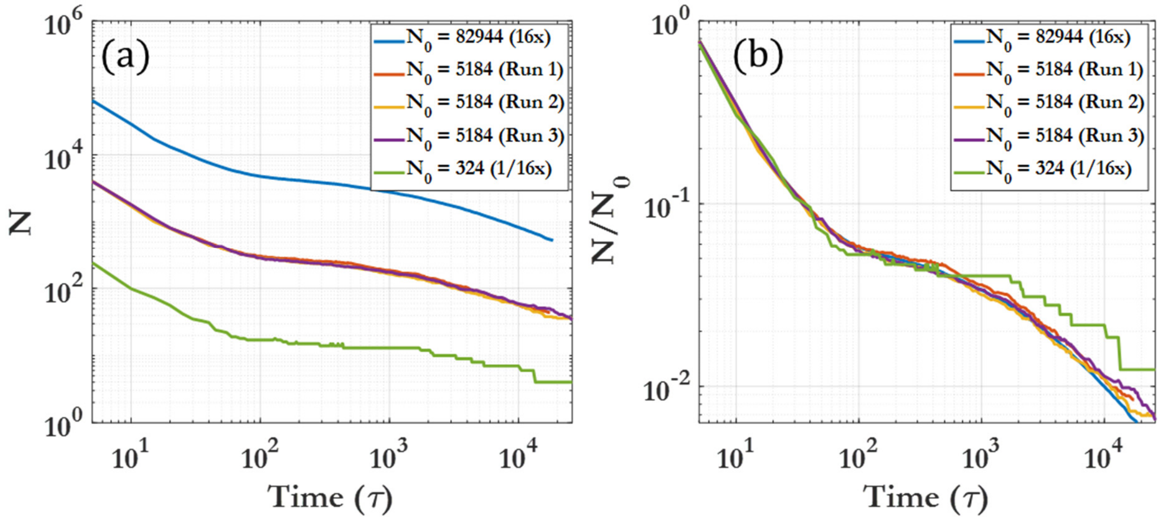

Figure S17: Size effects of the system. On clustering behavior. θ = 25% F = 0.

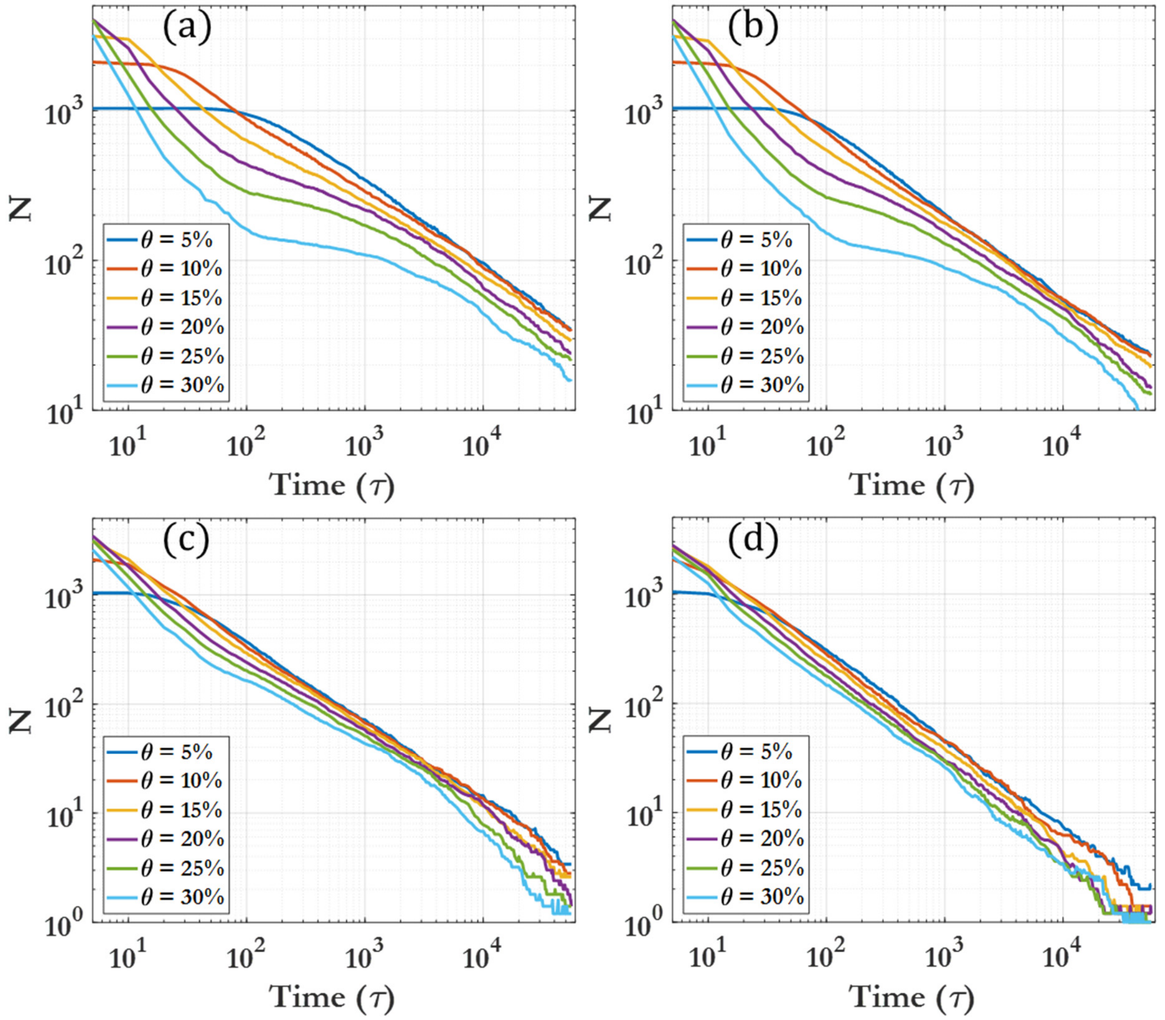

Figure S18: (a) Cluster number for F = 0, (b) F = 1, (c) F = 5, (d) F = 10.

References

[1] C.H. Rycroft, C.H. Wu, Y. Yu, K. Kamrin, Reference map technique for incompressible fluid-structure interaction, J Fluid Mech, 898 (2020).

[2] M. Theillard, D. Saintillan, Computational mean-field modeling of confined active fluids, J Comput Phys, 397 (2019).

[3] M. Tateno, H. Tanaka, Numerical prediction of colloidal phase separation by direct computation of Navier-Stokes equation, Npj Comput Mater, 5 (2019).

[4] M.P. Howard, A. Nikoubashman, J.C. Palmer, Modeling hydrodynamic interactions in soft materials with multiparticle collision dynamics, Curr Opin Chem Eng, 23 (2019) 34-43.

[5] G. Mirabello, A. Ianiro, P.H.H. Bomans, T. Yoda, A. Arakaki, H. Friedrich, G. de With, N.A.J.M. Sommerdijk, Crystallization by particle attachment is a colloidal assembly process, Nat Mater, 19 (2020) 391-+.

[6] S. Das, J. Riest, R.G. Winkler, G. Gompper, J.K.G. Dhont, G. Nagele, Clustering and dynamics of particles in dispersions with competing interactions: theory and simulation, Soft Matter, 14 (2018) 92-103.

[7] R. Singh, M.E. Cates, Hydrodynamically Interrupted Droplet Growth in Scalar Active Matter, Phys Rev Lett, 123 (2019).

[8] A. Paul, S. Mukherjee, J. Dhar, S. Ghosal, S. Chakraborty, The effect of the finite size of ions and Debye layer overspill on the screened Coulomb interactions between charged flat plates, Electrophoresis, 41 (2020) 607-614.

[9] W. Wang, W.T. Duan, S. Ahmed, A. Sen, T.E. Mallouk, From One to Many: Dynamic Assembly and Collective Behavior of Self-Propelled Colloidal Motors, Accounts Chem Res, 48 (2015) 1938-1946.

[10] J.T. Padding, A.A. Louis, Interplay between hydrodynamic and Brownian fluctuations in sedimenting colloidal suspensions, Phys Rev E, 77 (2008).

[11] J.T. Padding, A.A. Louis, Hydrodynamic interactions and Brownian forces in colloidal suspensions: Coarse-graining over time and length scales, Phys Rev E, 74 (2006).

[12] N. Yu, X. Lou, K. Chen, M.C. Yang, Phototaxis of active colloids by self-thermophoresis, Soft Matter, 15 (2019) 408-414.
